# An Exposition of the Signs and Symptoms of Pregnancy, the Period of Human Gestation, and the Signs of Delivery

**Published:** 1842-07

**Authors:** 


### ART. XX.

*Die Lehre von den Zeichen, Ersheinungen und der Dauer der menschlichen Schwangerschaft, so wie von den Phänomenen einer überstandenen Geburt.* Von W. F. MONTGOMERY, A.M. M.D. M.R.I.A. *Bonn.*

*An Exposition of the Signs and Symptoms of Pregnancy, the Period of Human Gestation, and the Signs of Delivery.* By W. F. MONTGOMERY, A.M. M.D. M.R.I.A. Translated by Dr. SCHWANN. Introduction by Dr. KILIAN, Professor of Midwifery at Bonn.

IN a former Number of this Journal*, we reviewed the original work from which this translation has been made, and expressed very fully our favorable opinion of its merits; since that period, four years have

* No. VIII. p. 467, Oct. 1837.

passed away, and public opinion, both in this country, in America,* and on the continent, has fully ratified our verdict.

Were anything wanting to add to the high opinion of this excellent work which we have always entertained, we should find it in the circumstance of its introduction to the German public under the auspices of such a judge as Kilian, one highly qualified by his own continued studies and practical experience to guide the public judgment aright in this branch of medicine.

The translation appears to us to be correct, and as literal as the spirit of the two languages would permit of, doing considerable credit to Dr. Schwann's literary powers.

It is to be regretted that those beautiful plates of the areola surrounding the nipple in the successive months of pregnancy, which adorn Dr. Montgomery's work, have not been copied into the translation; by omitting them, the translator has acted in a certain degree illiberally towards the author, because it is impossible for any description, howsoever lucid, to convey to the mind the impression at once transferred to it by those beautiful drawings. We were also surprised to find that the plate illustrative of the decidual cotyledons, a structure first described by Montgomery, has also been omitted; the same remark applies to this, as to the former omission, and we think this plate, containing one of the most original points in Montgomery's work, ought to have been inserted.

In our former review of the English work, we stated that we considered Dr. Montgomery's observations on the "application of auscultation to be good, but rather meager," his evident object being to render it the confirmatory or conclusive evidence where other signs were present, although in some occasional instances it alone would be sufficient to detect the existence of pregnancy. Dr. Kilian, in his introduction, has followed up this matter, and has entered fully into the signs given in pregnancy by the stethoscope, we fear, with rather too much accuracy; yet, having done so, he feels it requisite to state his doubts and difficulties. Amongst the first of these, he thinks that it is not every accoucheur who possesses that sharp and clear sense of hearing and of appreciating sounds necessary for the discriminating of difficult cases. It would be as rational to expect that every one should have equally good sight; "and we will here subscribe to the opinion of Henne, and we have had a sufficiently extensive experience to prove, that there is not more success to be obtained in auscultating the uterus than the chest." Such are the words of Kilian, a good and well known stethoscopist. We find Dr. Montgomery quoting Laennec to the same effect, with this difference, that that accurate observer was of opinion that, to form a correct judgment from the sounds given by the uterus required more care, and was beset with more difficulties than all those found in investigating the diseases of the chest. If the stethoscopic symptoms alone were to determine pregnancy in every case, in what inextricable confusion would the practitioner be involved, who should be consulted in such a case as that recorded at page 123 of Dr. Montgomery's work, where a large abdominal tumour gave rise to suspicion of pregnancy, of which several symptoms existed, especially a very distinct *placentary murmur*. Here, too strong credence in the stethoscope would have engendered error, and we should not envy

* Two editions have since been printed in America.

the practitioner who pinned his faith on the instrument if, when nine and ten months had passed by, and no appearance indicated approaching labour, he should begin to suspect that he had allowed his quickness of ear to deceive him, and that he had foolishly made use of but one sense, in place of employing all in the service of his patient and of his own reputation.

Dr. Kilian animadverts in very animated terms on the confidence felt by some persons in their powers of hearing in determining the death of the fœtus in utero, and expresses his opinion that the spirit of imitation may lead young and inexperienced persons to be too ready in the employment of the forceps and perforator, a privilege which should alone be enjoyed by the masters in the art. Pursuing the subject, he refers to Dr. Collins of Dublin, to whom he gives the title of the *most daring perforator of any period:* he (says Dr. Kilian) only perforates when he can no longer hear the beat of the fœtal heart; who might not copy him when Deisch, one of the correctest and greatest amongst modern authorities, is involved in the very same accusation, and rest content, like Collins and all enthusiastic friends of the stethoscope, of being perfectly satisfied with its evidence alone? From these and other passages, we find that Kilian agrees with Montgomery in the useful, but guarded and cautious, employment of this instrument, fully feeling its value, but not permitting it to usurp the place of the other physical signs and symptoms.

The speculum, in the opinion of Kilian, is of far less value than the stethoscope in discovering the different states of pregnancy, although its utility is unquestionable, and its employment indispensable, in investigating the various diseases and alterations which occur in the os tincæ and vaginal parietes. He adduces a curious fact, that this instrument, which has been but a few years introduced into general practice, was known and employed some centuries ago; and reference is made to the work of Paulus Ægineta, (Ed. Basil, 1538,) where the instrument is described under the name of Δίοπτρον, and its employment is termed Διοπτρισμὸς; and Dr. Kilian recommends that the words Dioptrum and Dioptrismus, as analogous to Catheter and Catheterismus, should be adopted into our medical nomenclature, as far more elegant than the indelicate term, speculum vaginæ. We warmly join in this recommendation.

One of the signs of pregnancy laid down by Jacquemin, and attested by Ricord, Kluge, Parent-Duchatelet, Lauer, and others who have had very extensive experience, viz. a bluish or livid colour of the vagina from the os externum to the os uteri, is spoken of by Kilian as one of the most constant signs of pregnancy; and the only thing wanting to make it one of the most valuable signs is to know if it be found in this state of the uterus only. Dr. Montgomery, after quoting the opinions of Kluge, Jacquemin, &c., states that nothing within his observation contradicts the accuracy of the sign under consideration. Duchatelet mentions that M. Jacquemin's accuracy in discovering pregnancy by this test was fully proved by a trial made for this purpose on no less a number than 4500 prostitutes.

Still Dr. Montgomery entertains doubts whether this sign, so constant in pregnancy, may not exist under other circumstances producing increased vascular determination or congestion of the genital organs; he says: " In a case lately seen, it became necessary to examine the vagina at the time of menstruation, and this purple hue of the mucous membrane was distinctly perceived.....It is also known that a common mode, long in use, of ascertaining whether certain of the lower animals are in a state fit for intercourse with the male, or in heat as it is called, is to examine the orifice and internal surface of the vagina, which, under such circumstances, is found almost inky dark." Mr. Cruikshank, in the Philosophical Transactions for 1797, p. 199, states that the method pursued by the feeders of rabbits for knowing when the female is in fit state for impregnation, is to turn up the tail and invert part of the vagina, when, if the animal be, as they term it, "in heat," the orifice and internal surface will then *be black as ink from the great derivation of blood to these parts.* "These considerations," says Dr. Montgomery, "must considerably modify the value of this test; but, nevertheless, should subsequent observations prove that healthy pregnancy is invariably accompanied by such an appearance becoming visible within the first or second month, the fact would certainly be one of the most important additions ever made to our means of making a correct diagnosis in cases of early pregnancy, and the more especially as it would be applicable to a period at which we have no other satisfactory means of discovering the existence of that condition, and might occasionally, under peculiar circumstances, be resorted to with propriety and advantage."

The ninth chapter, in which an examination of substances expelled from the uterus—early ova, moles, hydatids, membranes formed in dysmenorrhœa, and in other conditions of uterine derangement,—is given, has been peculiarly well rendered by the translator. The entire chapter is full of interest. Our author does not take upon himself to decide the question as to whether moles are to be considered as a product of conception or not, although it is evident he leans to the former opinion ; yet his cautious advice in giving evidence in a medico-legal point cannot be too much admired. Having cited a number of conflicting authorities on this subject, he says "he is not prepared to undertake to reconcile them, but it appears to him almost certain that much of the discordance has arisen from substances of very different characters having been indiscriminately classed together under the general term of moles, some of which were undoubtedly neither more nor less than diseased ova, or remnants of such, while others were as certainly either merely condensed coagula, or perhaps uterine polypi. Hence Mahon* appears perfectly justified in making the following remarks : "The existence of moles properly so called, is extremely doubtful, since they may all be referred to some one or other of the substances of which we have spoken ; viz. a placenta which had continued its growth, the fœtus having perished ; the degenerated remains of the afterbirth : coagulated blood ; sarcomatous tumours or polypi of the uterus. The first two cannot exist except after sexual intercourse ; the other three may be found independently of it. This is the distinction, which it is of the greatest importance to make in questions of legal medicine, that we may not without cause compromise the reputation of the unmarried girl, or the widow of irreproachable life and conduct." In this view the writer entirely coincides, and thinks the medical jurist would not be justifiable in pronouncing any such mass expelled from the uterus as proof of pregnancy, except he can detect in it either the fœtus or a part of it, or some other of the component structures of the

* Médecine Légale, tom. i. p. 274.

ovum; and even then, without further proof, "we must not," to use the words of Morgagni,* "immediately doubt the woman's chastity, since, as has been said above, the placentula might have remained in the uterus formerly, in an abortion that had not been much taken notice of;" which remark he makes in reference to cases in which portions of placenta appeared to have been a long time retained in utero, from which they were afterwards expelled in the form of moles when the women were advanced in life and many years widows,† as happened in the case related by Ambrose Paré,‡ in which a mole was retained seventeen years.

Dr. Montgomery states his opinion unequivocally, that uterine hydatids do not occur except after sexual intercourse, and as a consequence of impregnation; he never having met or heard of a case in which their presence was not accompanied or preceded by the usual symptoms of pregnancy. "It may not be amiss to notice here an argument from analogy, which has been brought forward against this view of the question, namely, that hydatids being formed in other situations, as the brain, &c., why may they not occur in the uterus also independently of any other circumstance, as intercourse or conception? To this I would reply, first, that the hydatids produced in the situations alluded to differ *toto cœlo* in their characters from those of the uterus; and secondly, that whenever hydatids are formed it is always in connexion with serous membranes which do not exist in the uterus until the ovum is deposited there, whose membranes are essentially serous." This reasoning is ingenious: however, in a medico-legal point of view, Dr. Montgomery thinks "it would be presumptuous and absurd to maintain that, because we had always found hydatids in connexion with one particular cause, there might not be some other also capable of producing them; and as there may be a doubt, we must let the accused have the benefit of that doubt.

In writing this notice of Dr. Schwann's translation, we feel we have not done full justice to Dr. Montgomery's work; but we did not like to touch on any of the subjects which had entered into our former review, in which we naturally selected those which we thought of the highest interest. The sound and correct practical remarks, which abound throughout the volume, must be of extreme value to the physician in every country, and we perfectly accord with Dr. Kilian in the sentiment expressed in his preface, "that in advising his friend Dr. Schwann to undertake its translation, he was conferring a boon on his native land; that the transplanting of this work to a German soil would afford additional means to the German physician for contending against error, confirming truth, exciting him to new discoveries, and bringing his information on those subjects nearer and nearer to perfection."

---

### Art. XXI.

*Principles of Surgery.* By James Syme, F.R.S.E., Professor of Clinical Surgery in the University of Edinburgh, and Surgeon to the Queen. Third Edition.—*London*, 1842. 8vo, pp. 505, with Plates.

Under the above title, Mr. Syme has published a work which has extended to three editions, a pretty conclusive proof that it is highly esti-

* Epist. xlviii. art. 13.

† See Mém. de l'Acad. Roy. des Sc. for 1735: Vallisneri, tom. ii. cit. p. 2, cap. ult.

‡ Lib. xxiv. chap. xl. xliii. p. 718.

